# Chitopentaose inhibits hepatocellular carcinoma by inducing mitochondrial mediated apoptosis and suppressing protective autophagy

**DOI:** 10.1186/s40643-020-00358-y

**Published:** 2021-01-05

**Authors:** Chunfeng Zhu, Mengyao Zhao, Liqiang Fan, Xuni Cao, Quanming Xia, Jiachun Zhou, Hao Yin, Liming Zhao

**Affiliations:** 1 School of Biotechnology, State Key Laboratory of Bioreactor Engineering, East China University of Science and Technology, No. 130 Meilong Road, Shanghai, 200237 China; 2 School of Life Sciences, Shandong University of Technology, Zibo, 255049 China; 3 Shanghai Collaborative Innovation Center for Biomanufacturing Technology (SCICBT), Shanghai, 200237 China; 4 Organ Transplant Center, Shanghai Changzheng Hospital, Shanghai, 200003 China

**Keywords:** Chitooligosaccharides, Singular DP, HCC, Apoptosis, Autophagy

## Abstract

Hepatocellular carcinoma (HCC) is one of the most prevalent and deadliest cancers. In this study, the anti-tumor effect of singular degree of polymerization (DP) chitooligosaccharides (COS) (DP 2–5) and the underlay molecular mechanisms were investigated on HCC cell line HepG2. MTT assay showed that (GlcN)_5_ have the best anti-proliferation effect among the different DP of COS (DP2-5). Furthermore, the administration of (GlcN)_5_ could decrease mitochondrial membrane potential, release cytochrome c into cytoplasm, activate the cleavage of Caspases9/3, thus inducing mitochondrial-mediated apoptosis in HepG2 cells (accounting for 24.57 ± 2.25%). In addition, (GlcN)_5_ treatment could increase the accumulation of autophagosomes. Further investigation showed that (GlcN)_5_ suppressed protective autophagy at the fusion of autophagosomes and lysosomes. Moreover, the inhibition of protective autophagy flux by (GlcN)_5_ could further decrease cell viability and increase the apoptosis rate. Our findings suggested that (GlcN)_5_ suppressed HepG2 proliferation through inducing apoptosis via the intrinsic pathway and impairing cell-protective autophagy. COS might have the potential to be an agent for lowering the risk of HCC.
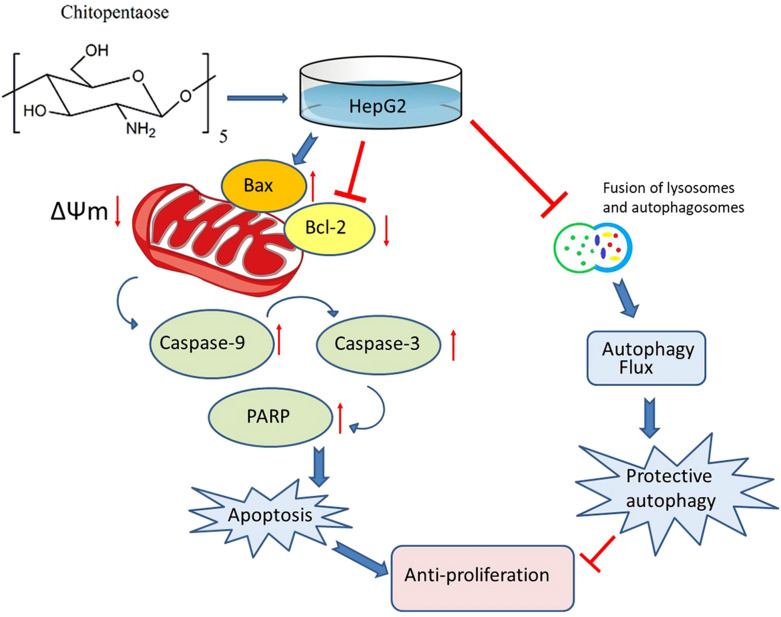

## Introduction

Hepatocellular carcinoma (HCC) ranks fifth in male cancer patients and the ninth in female cancer patients around the world, and it is characterized by a high mortality rate (Stewart and Wild 2014). Current treatments against HCC include liver transplantation, surgical resection, local ablation, and targeted molecular therapy such as Sorafinib, a multikinase inhibitor against vascular epithelial growth factor receptor-2/3 and raf-kinase (Rahbari et al. 2011). However, potentially curative therapies are restricted by liver source, patients’ disease stage, and liver function, and patients receiving targeted molecular therapy suffer from several side effects such as fatigue, diarrhea, and hand-foot skin reaction (Dong and Roberts 2010). In addition, drug resistance is another issue that highly affects the prognosis of HCC patients (Hwang et al. 2007). Therefore, searching for alternative effective agents with high safety, efficiency, and quality is urgently needed for improving the treatment for HCC.

Chitooligosaccharides (COS) is the hydrolysis product of chitosan, which exert multiple bioactivities including anti-inflammatory (Bahar et al. 2012), anti-microbials (Jumaa et al. 2002), anti-Alzheimer’s disease (Jia et al. 2016), anti-obesity (Li et al. 2018), anti-oxidative stress (Qiao et al. 2011), wound healing (Ueno et al. 1999), and improving plant defense response (He et al. 2018). Recently, the anti-cancer property of COS has received high attention. Previous studies had revealed that COS could inhibit the progression of several cancers including colon carcinoma, cervical cancer and gastric cancer (Luo et al. 2014; Zhao et al. 2019; Yuan et al. 2018). However, most of these studies were performed with the combination of COS with different degrees of polymerization (DP), and the anti-tumor effect of COS with singular DP was quite unclear.

Inducing apoptosis is a common strategy for inhibiting cancer progression (Wong 2011). Moreover, it was reported that the inhibition of autophagy could enhance apoptosis in HCC (Longo et al. 2008). Indeed, the crosstalk of autophagy and apoptosis impacts the progression of tumors and, therefore, is highly concerned (Eisenberg-Lerner et al. 2009). However, it remains unknown whether the crosstalk of apoptosis and autophagy is involved in the COS mediated anti-cancer effect, as well as how it works.

This research was designed to determine the effect of singular DP COS on HCC cell proliferation in vitro and explore the role of COS in the regulation of apoptosis and autophagy.

## Materials and methods

### Cell lines and reagents

Human liver epithelial HepG2 cell line was purchased from the Type Culture Collection of the Chinese Academy of Sciences (Shanghai, China). Fetal bovine serum (FBS) was purchased from Gibco (Gaithersburg, MD). Dulbecco’s Modified Eagle Medium (DMEM) was purchased from HyClone (Logan, UT). Antibodies against cleaved cysteinyl aspartate specific proteinase (caspase)-9 (1:1000), cleaved caspase-3 (1:1000), Bcl2 (1:1000), Bax (1:1000), β-actin (1:1000), and microtubule-associated proteins 1A/1B light chain 3B (LC3B) (1:1000) were obtained from Cell Signaling Technology (Danvers, MA). Anti-poly ADP-ribose polymerase-1 (anti-PARP-1) (1:1000) was provided from Abcam (Cambridge, UK). Anti- SQSTM1/p62 (1:1000), anti-Beclin1 (1:1000), and anti-lysosomal-associated membrane protein 1 (anti-LAMP1) (1:1000) were purchased from Proteintech (Rosemont, IL). 3-methyladenine (3-MA) and chloroquine (CQ) were purchased from MedChem Express (Princeton, NJ). 3-(4,5-dimethyl-2-thiazolyl)-2,5-diphenyl-2-*H*-tetrazolium bromide (MTT), dimethyl sulfoxide (DMSO), and other chemical agents were of the analytical grade.

### Characterization of COS

The separation of COS and high-performance liquid chromatography (HPLC) were described previously in our recent work (Li et al. 2018). The molecular weight of COS was detected using matrix-assisted laser desorption/ionization-time of flight mass spectrometry (MALDI-TOF–MS, Biosystems 4700 Proteomics Analyzer, Applied Biosystem Inc., Foster City, CA). 2,5-dihydroxybenzoic acid was used as the matrix. ^1^H nuclear magnetic resonance (NMR) was performed on Bruker Avance-600 NMR spectrometer (Billerica, MA). COS was dissolved in D_2_O. Fourier transform–infrared (FT-IR) spectrometer (Nicolet 6700, Nicolet Instrument Co., Madison, WI) was used to detect the structure of chitopentaose (GlcN)_5_.

### Cell culture and viability assay

HepG2 cells were cultured in DMEM supplemented with 10% FBS in a humidified atmosphere with 5% CO_2_ at 37  C. The viability of HepG2 cells was measured by MTT assay as described previously (Mosmann 1983). Briefly, cells were seeded into a 96-well plate at a density of 1 × 10^4^ per well for 24 h, and then exposed to different concentrations (1–7 mg/mL) of COS with different DP. Afterward, a solution containing 100 μg MTT was added to each well and incubated for 4 h at 37  C. After removing the solution, the formazan was dissolved with 150 μL of DMSO. The absorbance at 570 nm was read on a microplate reader (Tecan, Zürich, Switzerland). The cell viability was defined as the percentage of the control group. The calculated 50% inhibition concentration (IC_50_) value was calculated using Graphpad Prism (Ver. 7.0, Graphpad Software, La Jolla, CA) through non-linear regression.

### Determination of HepG2 apoptosis

#### Annexin V-fluorescein isothiocyanate isomer I (FITC)/propidium iodide (PI) dual staining

Annexin V-FITC/PI staining was conducted to detect the apoptotic cells. Briefly, cells were seeded in a 6-well plate at a density of 5 × 10^6^ per well overnight and then exposed to COS. After 48 h, cells were harvested and centrifuged at 1000*g* for 5 min and washed with phosphate buffer saline (PBS) once. Afterward, cells were suspended with 200 μL of staining buffer. 5 μL of Annexin V-FITC and 5 μL of PI were added and cells were incubated in the darkroom for 20 min. Finally, 300 μL of staining buffer was added. The apoptotic cells were determined using flow cytometry (BD Bioscience, Franklin Lakes, NJ).

#### DAPI staining and morphological analysis

Cells were seeded in a 24-well plate at a density of 1 × 10^5^ per well and treated with COS for 48 h. Culture medium was removed and cells were fixed with 4% (*v/v*) paraformaldehyde for 20 min at room temperature. After treatment, cells were stained with DAPI solution (Beyotime, Shanghai, China) for 20 min at room temperature. The nuclear morphological analysis was determined using an inverted fluorescence microscope (Nikon, Tokyo, Japan).

#### Detection of mitochondrial membrane potential

The mitochondrial membrane potential was measured using the JC-1 mitochondrial membrane potential (MMP) assay kit (Yeasen, Shanghai, China). In brief, HepG2 cells were seeded in a 96-well plate and treated with COS for different dosages for 48 h. JC-1 staining solution was added to each well and the cells were incubated at 37 °C for 20 min. Afterward, the supernatant was removed, and each well was washed twice. The red (excitation 490 nm, emission 530 nm) and green (excitation 525 nm, emission 590 nm) fluorescence were detected using a microplate reader. The MMP was represented as the ratio of green/red fluorescence.

### Determination of cell autophagy

#### Transmission electron microscopy (TEM)

Cells were planted in a 6-well-plate. After being incubated with or without CQ for 2 h, cells were treated with COS for 48 h. After being harvested, cells were fixed with 4% glutaraldehyde overnight at 4 °C and washed 3 times with PBS. Afterward, cells were fixed with 2% osmium tetroxide for 4 h at 4 °C and dehydrated with gradient ethanol. After being embedded and selected, the cells were stained with uranyl acetate and led acetate. The samples were observed using an electron microscope (JEOL, Tokyo, Japan).

#### Transfection of mRPF-GFP-LC3

The transfection of mRFP-GFP-LC3 and detection of the fusion of LC3 and lysosomes were performed using an mRFP-GFP-LC3 adenovirus vector kit (Hanbio, Shanghai, China) according to the manufacture’s instruction. Briefly, HepG2 cells were seeded in a 24-well plate and incubated with adenovirus to transfect the mRFP-GFP-LC3 vector for 6 h. After being treated with 3-MA or CQ for 2 h to block the autophagy flux, the cells were incubated with chitopentaose for 48 h. The fluorescence signals were detected using inverted fluorescence microscopy.

#### Colocalization of LC3 and lysosomes

Immunofluorescence was used to detect the colocalization of LC3 and lysosomes. Cells were seeded in a 24-well plate and pre-treated with or without CQ for 2 h and then treated with COS for 48 h. After removing the medium, cells were fixed with methanol for 20 min on ice and rinsed with PBS 3 times. The cells were blocked with PBS containing 5% FBS and 1% Triton 100 and incubated with primary antibody at 4 °C overnight. After being washed 3 times with PBS, the cells were incubated with FITC-labeled (for LC3 detection) or Alexa Fluor 594-labeled (for lysosome detection) secondary antibody (1:50) (Yeasen, Shanghai, China) for 1 h at room temperature. An anti-fade mounting medium (Beyotime, Shanghai, China) was used to preserve the fluorescence signals. The fluorescence signals were detected using an inverted fluorescence microscope. The colocalization analysis was conducted through ImageJ software (ver. 1.52a, Wayne Rasband, NIH). Pearson correlation coefficient was used to represent the colocalization of red and green fluorescence signals.

#### RNA extraction and quantitative polymerase chain reaction (qPCR) assays

Cells were seeded in a 6-well plate at a density of 5 × 10^6^ per well overnight and then exposed to chitopentaose for 48 h. RNA was extracted using an RNA extraction kit (Promega, Fitchburg, WI) according to the manufacturer’s guidance. Primers were synthesized through Sagon (Shanghai, China). Reverse transcription was conducted using a reverse transcription kit (Promega, Fitchburg, WI) according to the manufacture’s instruction. qPCR was conducted on a real-time thermal cycler (Bio-rad, Hercules, CA). The relative amount of target mRNA was calculated by the comparative cycle threshold (C_t_) method (2^−ΔΔCt^) by normalizing target mRNA C_t_ values to those for β-actin.

#### Protein extraction and western-blot

For total protein extraction, after being treated with different concentration of chitopentaose, HepG2 cells were lysed with a RIPA (Thermo, Waltham, MA) and 1 mM PMSF (Beyotime, Shanghai, China) cocktail using an ultrasonic cell disruptor. Separation of cytosolic protein and mitochondrial protein was performed using a cytosol-mitochondrial isolation kit (Beyotime, Shanghai, China). Protein concentration was analyzed using a BCA kit (Beyotime, China). An equal amount of protein was electrophoresed by 12% or 15% sodium laurylsulfonate (SDS) gel and transferred to polyvinylidene fluoride membrane by the wet transfer method (Bio-rad, Hercules, CA). The membrane was blocked with TBST (20 mM Tris-base, 150 mM NaCl, 0.1% Tween-20) containing 5% (*m/v*) non-fat milk powder and incubated with primary antibody at proper dilutions overnight at 4 °C. After being washed 3 times with TBST, the membrane was incubated with secondary antibody (1:3000) for 1 h at room temperature. The immunoblot on the membrane was developed with an enhanced chemiluminescence system. β-actin was used as an internal reference. The relative protein concentration was expressed as folds of the control group. The intensity of the immunoblot was quantified with ImageJ software.

### Statistical analysis

All the experiments were replicated more than three times. The data were expressed as mean ± standard deviation (SD). Statistical analysis was performed on SPSS (Ver. 22 SPSS Inc., Chicago, IL). One-way/two-way analysis of variance (ANOVA) and t-test were performed to evaluate the data. Significances were considered to exist at the level of *p* $<$ 0.05.

## Results and discussion

### ***Characterization of (GlcN)***_***5***_

**Fig. 1:**
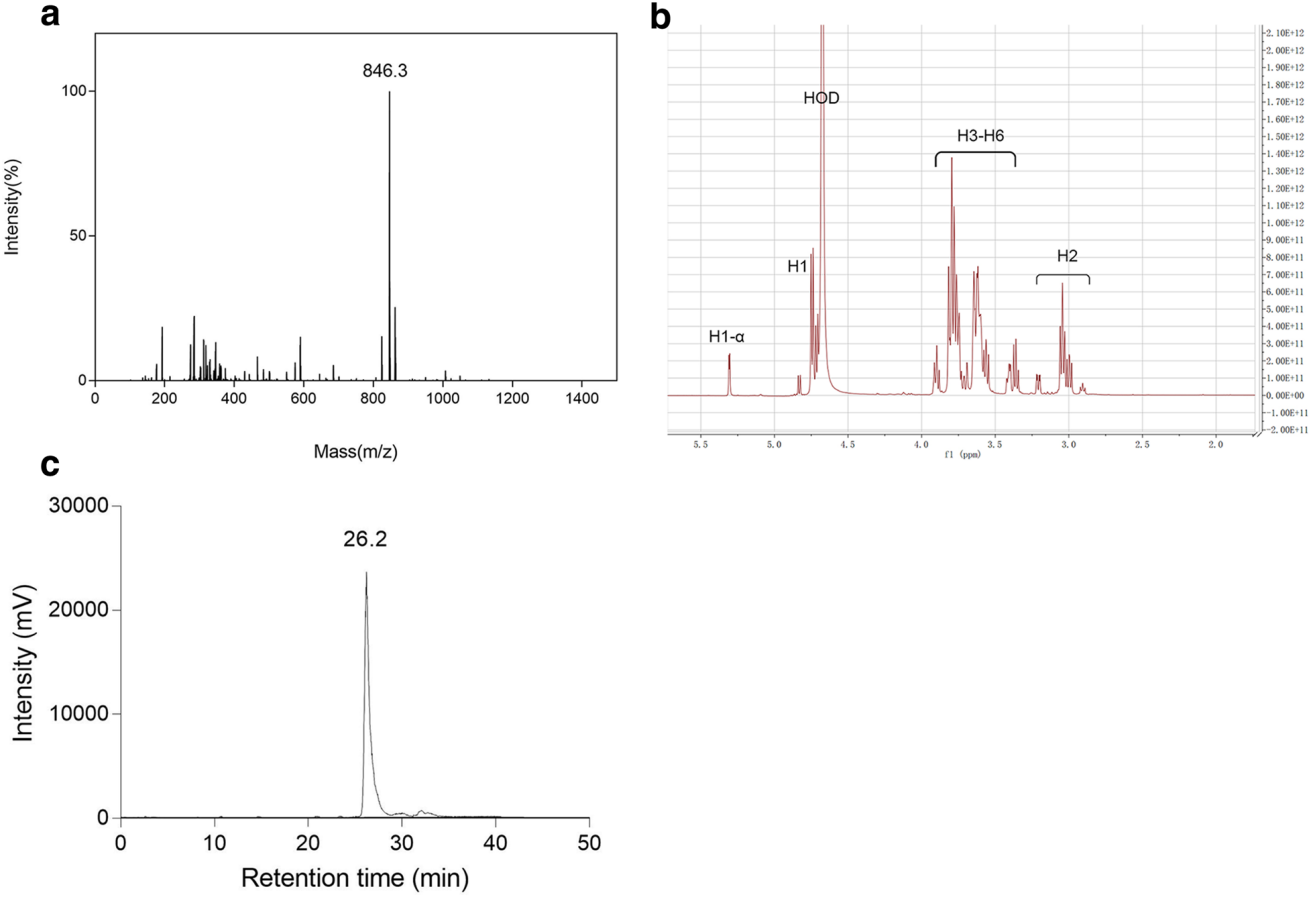
Structural characterization of (GlcN)_5_. **a** MALDI-TOF-MASS spectrometry analysis of (GlcN)_5_. **b** ^1^H NMR spectrometry analysis of (GlcN)_5_. **c** HPLC analysis of (GlcN)_5_

The result of the MALDI-TOF–MS analysis is shown in Fig. 1a. The mass/charge (*m/z*) of (GlcN)_5_ was 846.3 mass units corresponding to its [M + Na^+^] ion-peak, which was in line with a previous study (Chen et al. 2019). The results of the ^1^H NMR analysis is shown in Fig. 1b. There were absorbance peaks at 5.30 ppm (H-1α), 4.74 ppm (H-1), four absorbance peaks at 3.91–3.34 (H-3, H-4, H-5, H-6), absorbance peaks at 2.89–3.92 ppm (H-2), while no peak at 1.92 ppm (Acetyl-H) was found, which indicated that (GlcN)_5_ was highly deacetylated. The purity of (GlcN)_5_ was identified by the HPLC result (Fig. 1c), which showed one peak and the retention time was 26.2 min. These results indicated that (GlcN)_5_ was of high purity and degree of deacetylation.

### Anti-proliferation effect of COS

**Fig. 2:**
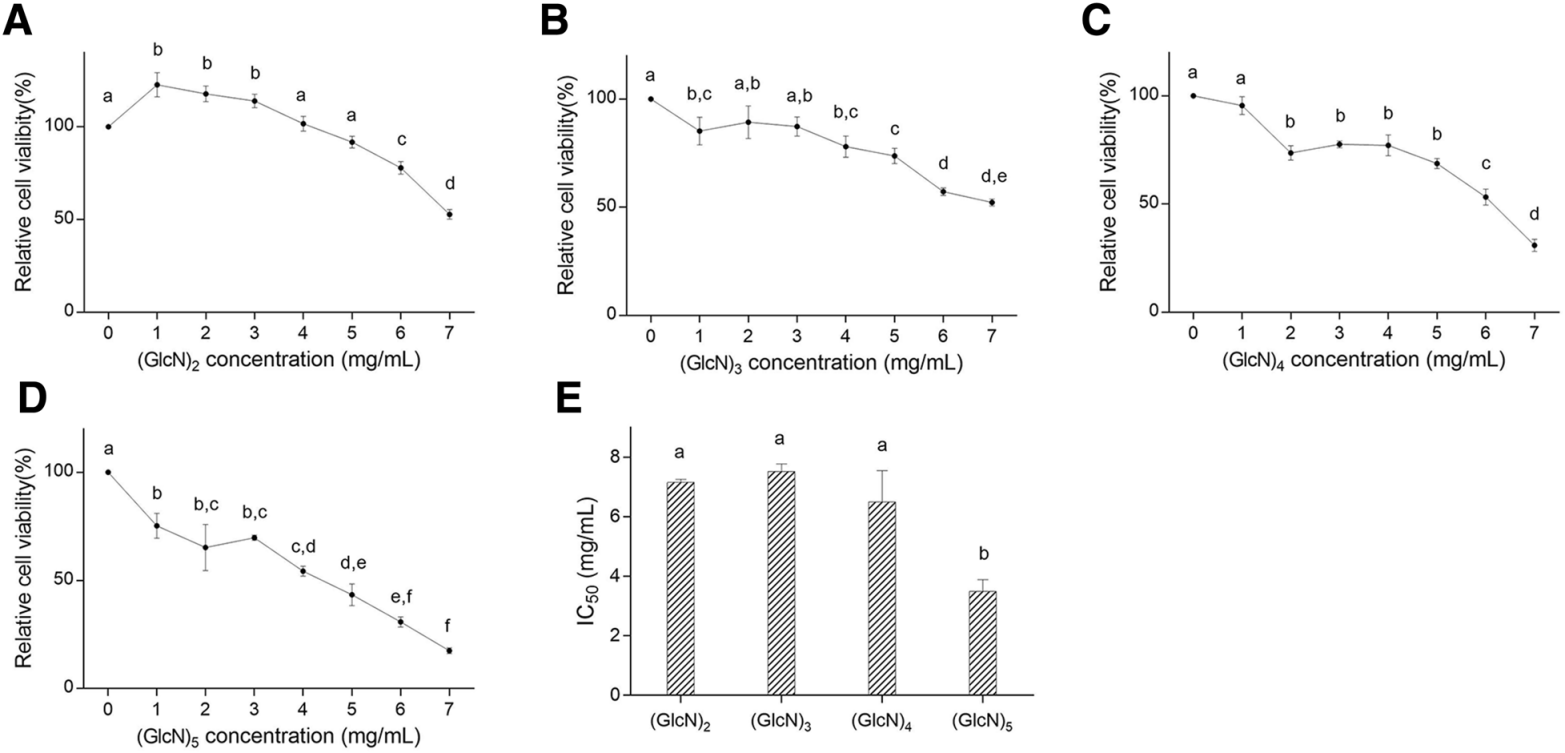
COS (DP 2–5) inhibited Cell viability in HepG2 cells. **A** (GlcN)_2_. **B** (GlcN)_3_. **C** (GlcN)_4_. **D** (GlcN)_5_. **E** IC_50_ values. Cells were treated with or without different concentration (0–7 mg/mL) of COS for 48 h. Data were represented as mean ± SD, significance between groups was marked with different letters, a, b, c, etc*.* (One-way ANOVA)

HepG2 cells were incubated with different concentrations of COS (DP 2–5) for 48 h. As shown in Fig. 2, the anti-proliferation effect of COS was showed in a dose- and DP-dependent manner. These data suggested that COS could suppress HepG2 proliferation, but each singular DP COS had a different inhibitory effect on HepG2. Various previous researches showed the anti-proliferation effect of COS on tumor cells. The relative cell viability when treated with 1 mg/mL COS for 48 h were reported ranging from 65 to 90% (Luo et al. 2014; Shen et al. 2009; Xu et al. 2008). All these reported generally used a complicated COS mixture (DP 2–9, for example (Xu et al. 2008)), which had different characteristic including the degree of N-acetylation (DA), DP, and the mean molecular weight. COS with lower DA, higher DP, and larger mean molecular weight showed better anti-proliferation effect on cancer cells (Kim et al. 2012), and our results further verified that COS with higher DP had a better inhibition effect on HepG2 cell proliferation, and (GlcN)_5_ showed better inhibitory effect on HepG2 cells compared with (GlcN)_1–4_. Besides, low concentration of (GlcN)_2_ promoted HepG2 cell proliferation, which could be explained by that: low concentration of (GlcN)_2_ might be used or enhance the synthesis of glycogen to promote HepG2 cell proliferation (Heni et al. 2011; Kirkman and Whelan 1986).

### COS induced apoptosis to HepG2 cells

**Fig. 3:**
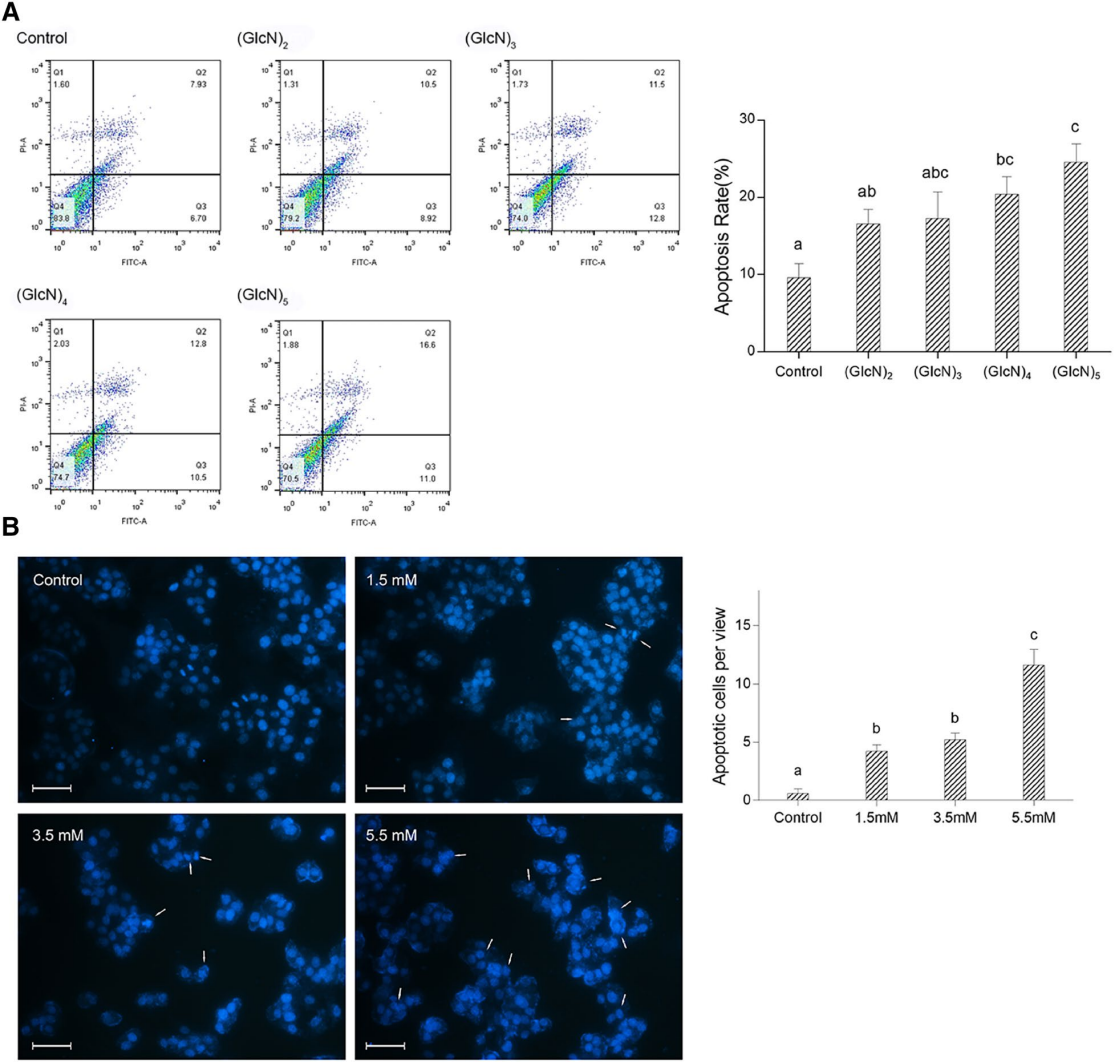
COS (DP 2–5) induced apoptosis in HepG2 cells. **A** Cells were treated with COS (DP 2–5) at the concentration of their respective IC_50_. Apoptotic cells (Q2 + Q3) were quantified. **B** Cells were treated with different concentration of (GlcN)_5_. Apoptotic cells per view were counted and quantified. Scale bar represents 150 μm. White arrows indicated apoptotic cells. Data were represented as mean ± SD Significance between groups was marked by various letters, a, b, c, etc. (One-way ANOVA)

The unbalance between cellular apoptosis and proliferation regulates the cancer pathological progression (Fong et al. 2006). Therefore, inducing apoptosis might be a strategy to inhibit tumor progression. To investigate whether COS would induce apoptosis, Annexin V-FITC/PI staining was conducted. The cross gate separated the cells into four groups: normal cells (FITC^−^/PI^−^), early apoptosis cells (FITC^+^/PI^−^), late apoptosis cells (FITC^+^/PI^+^), and mechanically damaged cells (FITC^−^/PI^+^). As shown in Fig. 3A, after being incubated with COS, the proportion of apoptosis cells (early apoptosis + late apoptosis) was increased with the increase of DP, and (GlcN)_5_ induced a significant increase in apoptotic cells (*p* $<$ 0.05), accounting for 24.57 ± 2.25%. Thus, the possible mechanism of the suppression activity was investigated using (GlcN)_5_ in the following research.

Chromatin condensation and DNA fragmentation are the hallmarks of apoptosis (Liu et al. 1998). As shown in Fig. 3B, the white arrows pointed out the cells underwent chromatin condensation or DNA fragmentation. Compared with the control group, (GlcN)_5_ induced morphological changes in cell nuclear in a dose-dependent manner. These findings suggested that COS could induce apoptotic phenotype in HepG2 cells. Several natural compounds were reported to increase apoptotic phenotype in tumor cells including nuclear condensation and, therefore, having the potential intervention effect against cancer (Hellmann et al. 2018; Salma et al. 2009).

### COS activate mitochondrial-caspase apoptosis

**Fig. 4:**
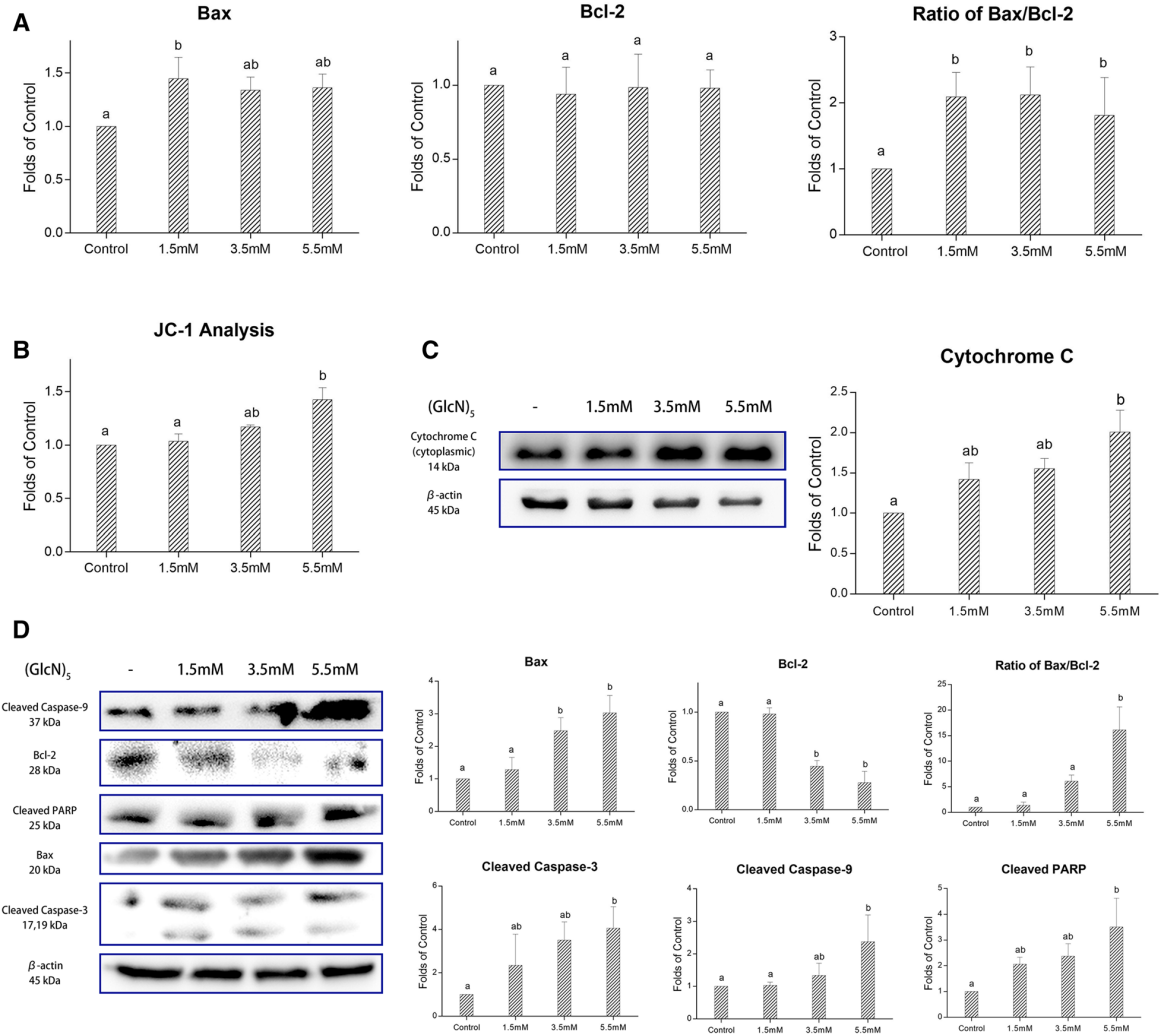
(GlcN)_5_ induced mitochondrial-mediated apoptosis in HepG2 cells. **A** Detection of bax and bcl-2 levels by qPCR. **B** Detection of mitochondrial membrane potential by JC-1 analysis. **C** Detection of cytoplasmic cytochrome c by western blot. **D** Detection of Bax, Bcl-2, cleaved Caspase-3, cleaved Caspase-9, and Cleaved PARP levels by western blot. Cells were treated with different concentration of (GlcN)_5_. Data were represented as mean ± SD Significance between groups was marked by various letters, a, b, c, etc. (One-way ANOVA)

Bcl-2 family proteins are highly involved in the progression of apoptosis (Mariadoss et al. 2019). The ratio of anti and pro-apoptosis protein in the Bcl-2 family is a key indicator to evaluate the effect of apoptosis stimuli. Real-time quantitative PCR was performed to detect the relative expression of bax and bcl-2. After being treated with (GlcN)_5_, the relative expression of the pro-apoptotic genes, bax, increased significantly (*p* $<$ 0.05), while the anti-apoptotic genes, bcl-2, showed no significant change. The ratio of bax/bcl-2 increased significantly (*p* $<$ 0.05) in a dose-dependent manner, indicating that cells underwent apoptosis (Fig. 4A). In line with the qPCR results, the relative expression of Bcl-2 decreased significantly, while Bax increased significantly after being treated with (GlcN)_5_. Thus, the ratio of Bax/Bcl-2 increased significantly (*p* $<$ 0.05) using western blot confirmation (Fig. 4D), suggesting that cells treated with (GlcN)_5_ showed an apoptotic tendency.

A decrease in MMP would result in the structural change in mitochondria and consequently leads to the redistribution of cytochrome c (Gottlieb et al. 2003; Mariadoss et al. 2020). JC-1 staining kit was used to examine the change of MMP. As shown in Fig. 4B, the ratio of green/red fluorescence decreased significantly with a dose-dependent manner, suggesting MMP was decreased after (GlcN)_5_ treatment. The release of cytochrome c from mitochondria to the cytoplasm was also detected by western blot. As shown in Fig. 4C, the relative expression of cytoplasmic cytochrome c of (GlcN)_5_ groups were significantly increased compared with the control group (*p* < 0.05). In line with a previous study, a polypeptide extracted from *Ciona savignyi* had a similar effect with (GlcN)_5_ that depolarized mitochondria, released cytochrome c into cytoplasm, and induced apoptosis (Cheng et al. 2012).

The release of cytochrome c from mitochondria to cytoplasm could activate pro-caspase-9, which consequently cleaves other caspase family proteins and finally executes cell to apoptosis (Vijayakumar et al. 2020; Vijayalakshmi et al. 2019). As shown in Fig. 4D, the relative expression of cleaved caspase-3, cleaved caspase-9, and cleaved PARP increased significantly (*p* < 0.05) when cells were treated with (GlcN)_5_. These findings above revealed that (GlcN)_5_ could alter the expression of Bcl-2 family protein, decrease the mitochondrial membrane potential, release cytochrome c from mitochondria to the cytoplasm, led to the cleavage of caspase-3 and caspase-9, which cleavage PARP, and finally induced apoptosis in HepG2 cells. Xu et al. reported similar results, that COS could increase the cleavage of PARP (Xu et al. 2008), however, whether the proteins in the caspase family were involved in the cleavage of PARP was not illustrated in their study. In this paper, we demonstrated that (GlcN)_5_ induced PARP cleavage in HepG2 cells via the mitochondria-caspase pathway.

### COS induced autophagy markers

**Fig. 5:**
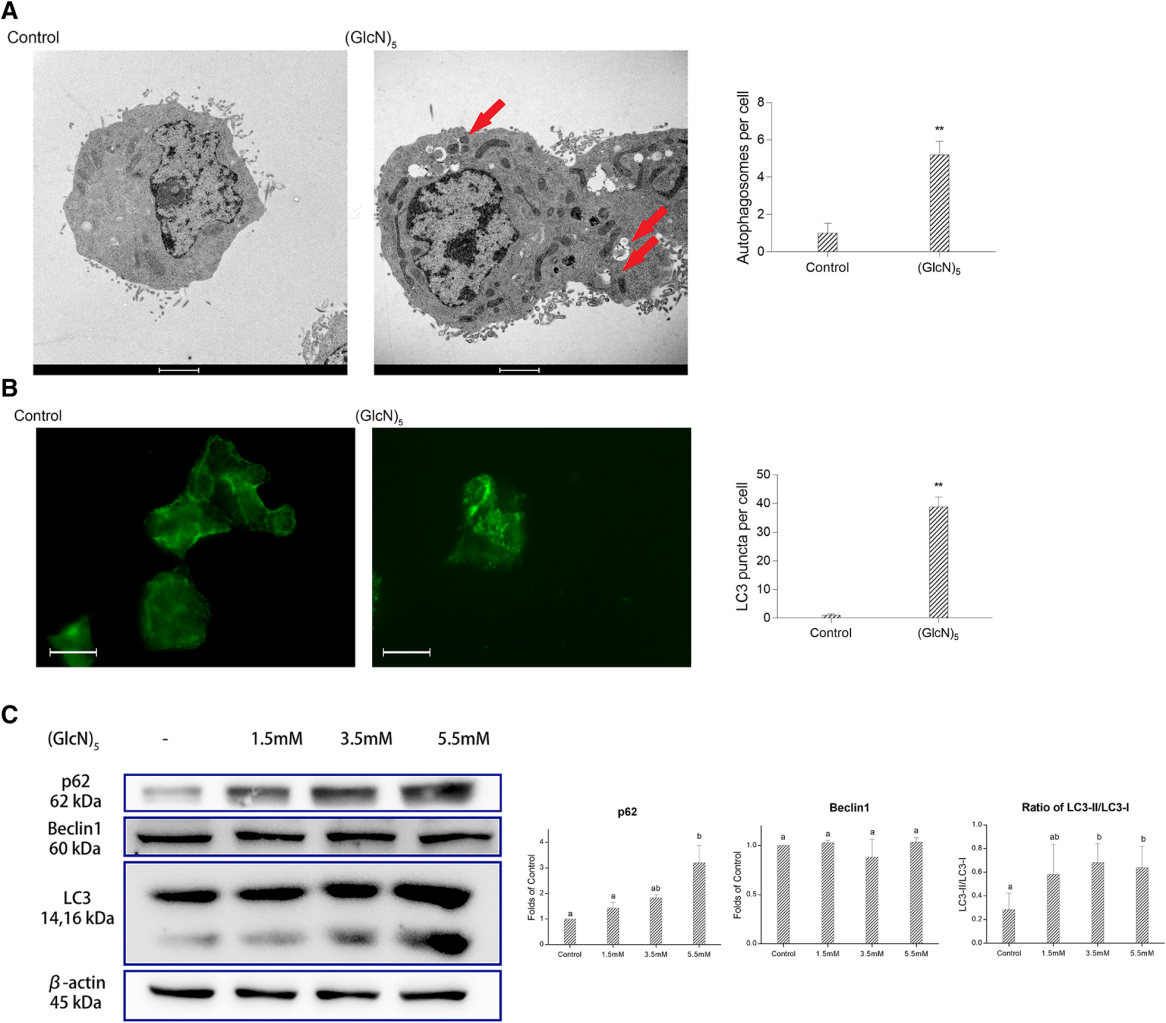
(GlcN)_5_ increased autophagy markers. **A** Representative image of TEM. Scale bar represents 2 μm. Autophagosomes per cell were counted and quantified. **B** Immunofluorescence results for LC3 puncta detection. Scale bar represents 50 μm. LC3 puncta per cell were counted and quantified. **C** Western blot analysis for p62, Beclin1, and LC3B. Cells were treated with different concentration of (GlcN)_5_. Cells were treated with or without (GlcN)_5_ (3.5 mM). Data were represented as mean ± SD Significance between groups was marked by various letters, a, b, c, etc. (One-way ANOVA). **, *p* < 0.01, ***, *p* < 0.001 (t-test)

Autophagy plays a dual role during the physiological process and tumorigenesis including maintenance of cell survival and the defeat of cell malfunction (Hippert et al. 2006; Mizushima 2007). It was reported that the inducing (Yo et al. 2009) and suppression (Law et al. 2014) of autophagy could both inhibit tumor cell viability. In the present study, we further investigated the effect of (GlcN)_5_ on autophagy in HepG2 cells. TEM is a golden standard to detect the formation of autophagosomes. As shown in Fig. 5A, after being treated with (GlcN)_5_, HepG2 showed an increase in the formation of autophagosomes compared to the control group. Meanwhile, the markers of autophagy include the conversion of LC3-I to LC3-II which indicates the accumulation of autophagosomes. In the present study, after cells being treated with (GlcN)_5_, the green dots increased compared with control group, suggesting that (GlcN)_5_ could accumulate autophagosomes (Fig. 5B). These data were consistent with the blots of LC3B (Fig. 5C), in which LC3-II expression increased in a dose-dependent manner after (GlcN)_5_ treatment. In addition, the Beclin1 showed no significant change, and the p62 blots increased significantly (*p* $<$ 0.05) compared with control group, which suggested that the (GlcN)_5_ treatment might have no effect on the formation of autophagosomes but suppress the degradation of autophagosomes.

### COS suppressed autophagy flux

The accumulation of autophagosomes might be caused by the increase of the formation of autophagosomes or by the reduction of the degradation of autophagosomes (Meng et al. 2018). To further investigate these two possibilities, two chemical inhibitors were co-incubated with (GlcN)_5_: CQ, an inhibitor of the fusion of autophagosomes and lysosomes, and 3-MA, an inhibitor of the formation of autophagosomes.

**Fig. 6:**
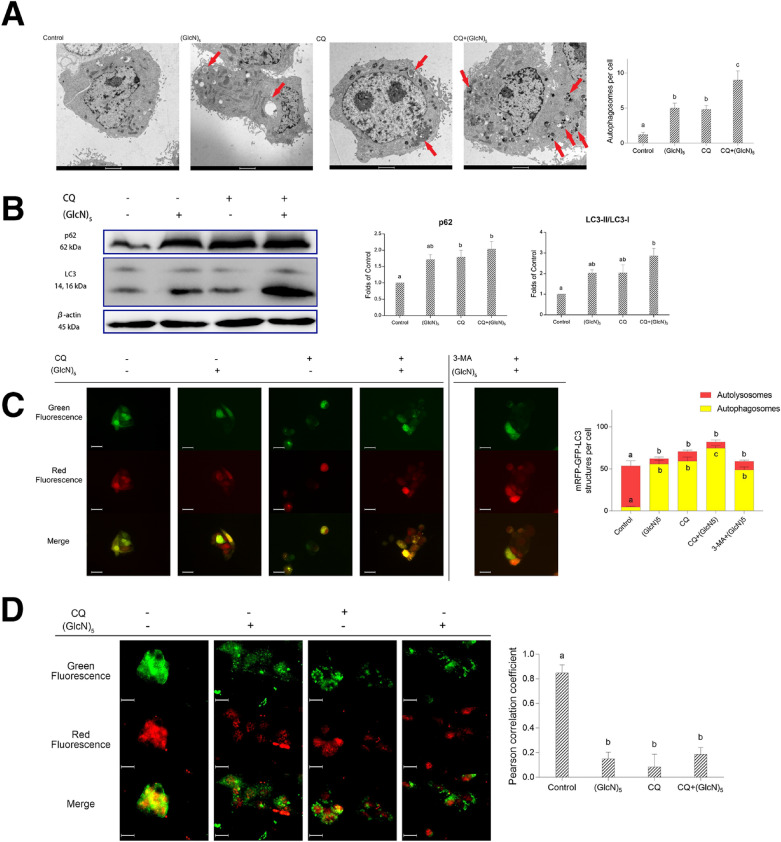
(GlcN)_5_ suppressed autophagy flux. **A** TEM results. Scale bar represents 2 μm. Autophagosomes per cell were counted and quantified. **B** Western blot analysis for p62 and LC3B. **C** LC3 puncta detection through transfection of mRFP-GFP-LC3. Scale bar represents 50 μm. LC3 puncta per cell were counted and quantified. **D** Colocalization analysis for autophagosomes and lysosomes. Scale bar represents 50 μm. Cells were treated with or without (GlcN)_5_ (5 mM), CQ (5 μM), or 3-MA (5 mM). Data were represented as mean ± SD Significance between groups was marked by various letters, a, b, c, etc. (One-way ANOVA)

The TEM results showed that in comparison with the control group, the formation of autophagosomes increased in HepG2 cells treated with CQ alone group and (GlcN)_5_ group (Fig. 6A). Moreover, the co-incubation of CQ and (GlcN)_5_ group induced an increase of the numbers of autophagosomes, suggesting (GlcN)_5_ might have a similar effect with CQ on autophagy, which suppressed the degradation of autophagosomes. This was further confirmed by the western blot assay. As shown in Fig. 6B, the p62 and LC3-II blot increased as the administration of CQ, almost the same as (GlcN)_5_ group, when compared to the control group, while the increase was even more significant (*p* < 0.05) when the cells were co-incubated with CQ and (GlcN)_5_.

Furthermore, we checked the autophagic flux after CQ and/or (GlcN)_5_ treatment using the mRFP-GFP-LC3 vector system in HepG2 cells. In this study, the LC3 proteins were labeled with red and green fluorescence protein, respectively. When the fusion of lysosomes and autophagosomes occurs, the green fluorescence quenches due to the decrease of pH in lysosomes and only the red dots remain. Conversely, yellow dots (the merge of green dots and red dots) emerged when the autophagy flux was suppressed at the late stage. As shown in Fig. 6C, the treatment of (GlcN)_5_ led to an increase of yellow dots, which was consistent with the CQ group, indicating the suppression of autophagy flux at the late stage. The co-incubation of CQ and (GlcN)_5_ resulted in a significant (*p* < 0.05) increase in the yellow dots when compared with the control group, CQ group, and (GlcN)_5_ group, showing a synergistic effect. In addition, 3-MA could not inhibit the accumulation of yellow dots when co-treated with (GlcN)_5_, suggesting that (GlcN)_5_ might not increase the formation of autophagosomes. To further explore whether (GlcN)_5_ impair the fusion of autophagosomes and lysosomes, colocalization analysis of LC3 puncta and lysosomes was conducted by immunofluorescence. LC3B proteins were labeled with green fluorescence, representing autophagosomes, and LAMP1 proteins were labeled with red fluorescence, representing lysosomes. As shown in Fig. 6D, with the treatment of (GlcN)_5_, the Pearson correlation coefficient of LC3B and LAMP1 decreased significantly (*p* < 0.05), indicating low colocalization. Similar situations were found in the CQ group and co-administration group (CQ + (GlcN)_5_). These results suggest (GlcN)_5_ inhibited the fusion of lysosomes and autophagosomes and impaired autophagy flux at the late stage, finally suppressed the autophagy in HepG2 cells.

### The role of autophagy in cell viability and apoptosis

**Fig. 7:**
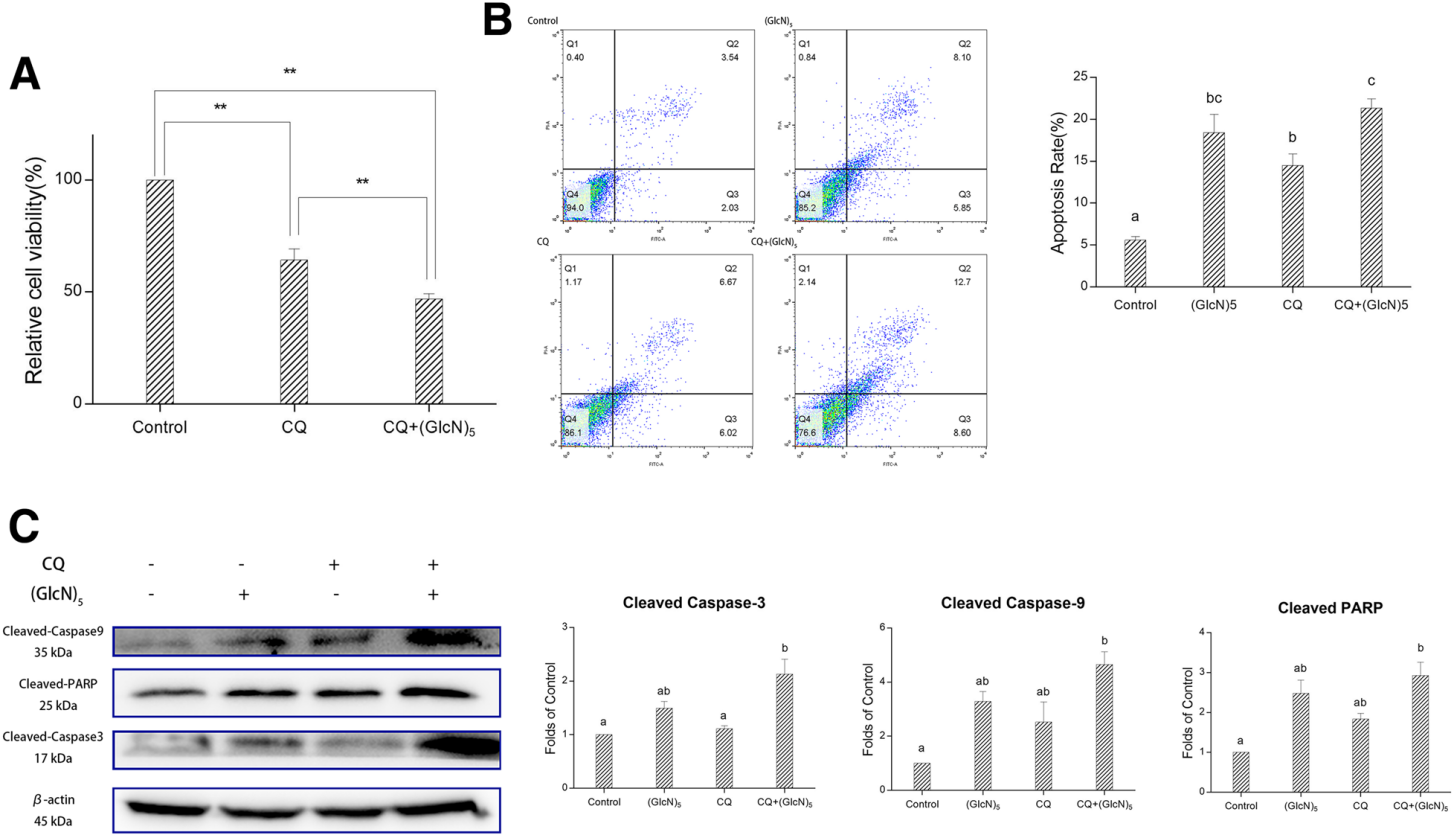
(GlcN)_5_ impair autophagy flux and induce apoptosis. **A** Cell viability detection. **B** Apoptosis analysis using flow cytometry. **C** Western blot analysis for cleaved Caspase-3, Caspase-9, and PARP. Cells were treated with or without (GlcN)_5_ (5 mM), or CQ (5 μM). Data were represented as mean ± SD, significance between groups was marked by various letters, a, b, c, etc*.* (One-way ANOVA). ***, *p* $<$ 0.001 (t-test)

The role of autophagy on tumor progression varies according to cell type and cancer stage (Glick et al. 2010). On one hand, autophagy prevents tumorigenesis by reducing inflammation and necrosis. On the other hand, it also protects the tumor by promoting cell viability under stress. In this study, we further evaluated the crosstalk between autophagy and apoptosis in HepG2, as well as investigated the role of autophagy in cell viability and apoptosis. As shown in Fig. 7A, cell viability decreased significantly (*p* < 0.01) with the CQ treatment. The co-incubation of CQ and (GlcN)_5_ suppressed the proliferation of HepG2 cells significantly (*p* < 0.01) when compared with the CQ group and (GlcN)_5_ group, suggesting the role of autophagy was to protect HepG2 cells from death. In accordance with the annexin V-FITC/PI dual staining assay (Fig. 7B), the co-incubation of CQ and (GlcN)_5_ induced a significant apoptotic phenotype, suggesting the inhibition of autophagy induced apoptosis. In western blot assay for further substantiation (Fig. 7C), the co-incubation of CQ and (GlcN)_5_ induced a significant increase (*p* < 0.05) in the relative expression of cleaved caspase-3, cleaved caspase-9, and cleaved PARP. These results indicated that the suppression of protective autophagy by (GlcN)_5_ in HepG2 cells could further lead to apoptosis and inhibition of cell proliferation.

## Conclusions

COS treatment suppressed the proliferation of HepG2 tumor cells through inducing apoptosis. Among the four kinds of singular DP COS, (GlcN)_5_ exerted the best effect on suppressing proliferation and inducing apoptosis. Further investigation revealed that (GlcN)_5_ could increase Bax expression and decrease Bcl-2 expression both in mRNA and protein level. Moreover, (GlcN)_5_ reduced the mitochondrial membrane potential, and released cytochrome c, thus inducing apoptosis via the intrinsic apoptosis pathway. In addition, (GlcN)_5_ could block the fusion of lysosomes and autophagosomes to impair autophagy flux, which further suppressed the proliferation but promoted apoptosis in HepG2 cells. Our findings indicated that (GlcN)_5_ could be a potential therapeutic agent for HCC. However, studies on the inhibition effect of (GlcN)_5_ on various tumor cells and in vivo studies should be conducted to further substantiation.

## Data Availability

All data generated or analyzed during this study are included in this published article.

## Abbreviations

HCC: Hepatocellular carcinoma
COS: Chitooligosaccharides
DP: Degree of polymerization
FBS: Fetal bovine serum
DMEM: Dulbecco’s Modified Eagle Medium
caspase: Cysteinyl aspartate specific proteinase
LC3B: Microtubule-associated proteins 1A/1B light chain 3B
PARP: Poly ADP-ribose polymerase
LAMP: Lysosomal-associated membrane protein
3-MA: 3-Methyladenine
CQ: Chloroquine
MTT: 3-(4,5-Dimethyl-2-thiazolyl)-2,5-diphenyl-2-*H*-tetrazolium bromide
DMSO: Dimethyl sulfoxide
HPLC: High-performance liquid chromatography
MALDI-TOF–MS: Matrix-assisted laser desorption/ionization-time of flight mass spectrometry
NMR: Nuclear magnetic resonance
FT-IR: Fourier transform–infrared
(GlcN)5: Chitopentaose
IC_50_: Calculated 50% inhibition concentration
FITC: Fluorescein isothiocyanate isomer I
PI: Propidium iodide
PBS: Phosphate buffer saline
MMP: Mitochondrial membrane potential
TEM: Transmission electron microscopy
SD: Standard deviation
ANOVA: Analysis of variance

## References

[CR1] Bahar B, Doherty JV, Maher S, McMorrow J, Sweeney T (2012). Chitooligosaccharide elicits acute inflammatory cytokine response through AP-1 pathway in human intestinal epithelial-like (Caco-2) cells. Mole Immunol.

[CR2] Chen P, Zhao M, Chen Q, Fan L, Gao F, Zhao L (2019). Absorption characteristics of chitobiose and chitopentaose in the human intestinal cell line Caco-2 and everted gut sacs. J Agric Food Chem.

[CR3] Cheng L, Wang C, Liu H, Wang F, Zheng L, Zhao J, Chu E, Lin X (2012). A novel polypeptide extracted from Ciona savignyi induces apoptosis through a mitochondrial-mediated pathway in human colorectal carcinoma cells. Clin Colorectal Cancer.

[CR4] Dong YJ, Roberts LR (2010). Hepatocellular carcinoma: a global view. Nat Rev Gastroenterol Hepatol.

[CR5] Eisenberg-Lerner A, Bialik S, Simon H-U, Kimchi A (2009). Life and death partners: apoptosis, autophagy and the cross-talk between them. Cell Death Differ.

[CR6] Fong P, Xue W, Ngan H, Chiu P, Chan K, Tsao S, Cheung A (2006). Caspase activity is downregulated in choriocarcinoma: a cDNA array differential expression study. J Clin Pathol.

[CR7] Glick D, Barth S, Macleod KF (2010). Autophagy: cellular and molecular mechanisms. J Pathol.

[CR8] Gottlieb E, Armour SM, Harris MH, Thompson CB (2003). Mitochondrial membrane potential regulates matrix configuration and cytochrome c release during apoptosis. Cell Death Differ.

[CR9] He YQ, Bose SK, Wang WX, Jia XC, Lu H, Yin H (2018). Pre-harvest treatment of chitosan oligosaccharides improved strawberry fruit quality. Int J Mol Sci.

[CR10] Hellmann MD, Ciuleanu TE, Pluzanski A, Lee JS, Otterson GA, Audigier-Valette C, Elisa M, Helena L, Sjaak B, Paz-Ares L (2018). Nivolumab plus Ipilimumab in lung cancer with a hgh tumor mutational burden. N Engl J Med.

[CR11] Heni M, Hennige AM, Peter A, Siegel-Axel D, Ordelheide A-M, Krebs N, Machicao F, Fritsche A, Haring H, Staiger H (2011). Insulin promotes glycogen storage and cell proliferation in primary human astrocytes. PLoS ONE.

[CR12] Hippert MM, O'Toole PS, Thorburn A (2006). Autophagy in cancer: good, bad, or both?. Can Res.

[CR13] Hwang IT, Chung YM, Kim JJ, Chung JS, Kim BS, Kim HJ, Kim JS, Do Yoo Y (2007). Drug resistance to 5-FU linked to reactive oxygen species modulator 1. Biochem Biophys Res Commun.

[CR14] Jia SL, Lu Z, Gao ZL, An J, Wu XL, Li XX, Dai XL, Zheng QS, Sun YX (2016). Chitosan oligosaccharides alleviate cognitive deficits in an amyloid-beta(1–42)-induced rat model of Alzheimer's disease. Int J Biol Macromol.

[CR15] Jumaa M, Furkert FH, Muller BW (2002). A new lipid emulsion formulation with high antimicrobial efficacy using chitosan. Eur J Pharm Biopharm.

[CR16] Kim E-K, Je J-Y, Lee S-J, Kim Y-S, Hwang J-W, Sung S-H, Moon SH, Jeon BT, Kim SK, Jeon Y-J (2012). Chitooligosaccharides induce apoptosis in human myeloid leukemia HL-60 cells. Bioorg Med Chem Lett.

[CR17] Kirkman BR, Whelan WJ (1986). Glucosamine is a normal component of liver glycogen. FEBS Lett.

[CR18] Law BYK, Chan WK, Su WX, Jing RW, Li PB, Liang L, Wong VKW (2014). Natural small-molecule enhancers of autophagy induce autophagic cell death in apoptosis-defective cells. Sci Rep.

[CR19] Li X, Zhao M, Fan L, Cao X, Chen L, Chen J, Lo., Y. M., & Zhao, L.  (2018). Chitobiose alleviates oleic acid-induced lipid accumulation by decreasing fatty acid uptake and triglyceride synthesis in HepG2 cells. J Funct Foods.

[CR20] Liu X, Li P, Widlak P, Zou H, Luo X, Garrard WT, Wang X (1998). The 40-kDa subunit of DNA fragmentation factor induces DNA fragmentation and chromatin condensation during apoptosis. Proc Natl Acad Sci.

[CR21] Longo L, Platini F, Scardino A, Alabiso O, Vasapollo G, Tessitore L (2008). Autophagy inhibition enhances anthocyanin-induced apoptosis in hepatocellular carcinoma. Mol Cancer Ther.

[CR22] Luo Z, Dong X, Ke Q, Duan Q, Shen L (2014). Downregulation of CD147 by chitooligosaccharide inhibits MMP-2 expression and suppresses the metastatic potential of human gastric cancer. Oncol Lett.

[CR23] Mariadoss AVA, Vinayagam R, Senthilkumar V, Paulpandi M, Murugan K, Xu B, Gothandam KM, Kotakadi VS, David E (2019). Phloretin loaded chitosan nanoparticles augments the pH-dependent mitochondrial-mediated intrinsic apoptosis in human oral cancer cells. Int J Biol Macromol.

[CR24] Mariadoss AVA, Saravanakumar K, Sathiyaseelan A, Venkatachalam K, Wang M-H (2020). Folic acid functionalized starch encapsulated green synthesized copper oxide nanoparticles for targeted drug delivery in breast cancer therapy. Int J Biol Macromol.

[CR25] Meng D, Li Z, Wang G, Ling L, Wu Y, Zhang C (2018). Carvedilol attenuates liver fibrosis by suppressing autophagy and promoting apoptosis in hepatic stellate cells. Biomed Pharmacother.

[CR26] Mizushima N (2007). Autophagy: process and function. Genes Dev.

[CR27] Mosmann T (1983). Rapid colorimetric assay for cellular growth and survival: application to proliferation and cytotoxicity assays. J Immunol Methods.

[CR28] Qiao Y, Bai X-F, Du Y-G (2011). Chitosan oligosaccharides protect mice from LPS challenge by attenuation of inflammation and oxidative stress. Int Immunopharmacol.

[CR29] Rahbari NN, Mehrabi A, Mollberg NM, Müller SA, Koch M, Büchler MW, Weitz J (2011). Hepatocellular carcinoma: current management and perspectives for the future. Ann Surg.

[CR30] Salma Y, Lafont E, Therville N, Carpentier S, Bonnafé M-J, Levade T, Génisson Y, Andrieu-Abadie N (2009). The natural marine anhydrophytosphingosine, Jaspine B, induces apoptosis in melanoma cells by interfering with ceramide metabolism. Biochem Pharmacol.

[CR31] Shen K-T, Chen M-H, Chan H-Y, Jeng J-H, Wang Y-J (2009). Inhibitory effects of chitooligosaccharides on tumor growth and metastasis. Food Chem Toxicol.

[CR32] Stewart, B., & Wild, C. P. (2014). World cancer report 2014.

[CR33] Ueno H, Yamada H, Tanaka I, Kaba N, Matsuura M, Okumura M, Kadosawa T, Fujinaga T (1999). Accelerating effects of chitosan for healing at early phase of experimental open wound in dogs. Biomaterials.

[CR34] Vijayakumar S, Vinayagam R, Anand MAV, Venkatachalam K, Saravanakumar K, Wang M-H, Gothandam K, David E (2020). Green synthesis of gold nanoparticle using Eclipta alba and its antidiabetic activities through regulation of Bcl-2 expression in pancreatic cell line. J Drug Deliv Sci Technol.

[CR35] Vijayalakshmi S, Mariadoss AVA, Ramachandran V, Shalini V, Agilan B, Sangeetha CC, Balu P, Kotakadi VS, Karthikkumar V, Ernest D (2019). Polydatin encapsulated poly [lactic-co-glycolic acid] nanoformulation counteract the 7, 12-dimethylbenz [a] anthracene mediated experimental carcinogenesis through the inhibition of cell proliferation. Antioxidants.

[CR36] Wong RS (2011). Apoptosis in cancer: from pathogenesis to treatment. J Exp Clin Cancer Res.

[CR37] Xu Q, Dou J, Wei P, Tan C, Yun X, Wu Y, Bai X, Ma X, Du Y (2008). Chitooligosaccharides induce apoptosis of human hepatocellular carcinoma cells via up-regulation of Bax. Carbohyd Polym.

[CR38] Yo YT, Shieh GS, Hsu KF (2009). Licorice and Licochalcone-A Induce Autophagy in LNCaP Prostate Cancer Cells by Suppression of Bcl-2 Expression and the mTOR Pathway. J Agric Food Chem.

[CR39] Zhao M, Gu L, Li Y, Chen S, You J, Fan L, Wang Y, Zhao L (2019). Chitooligosaccharides display anti-tumor effects against human cervical cancer cells via the apoptotic and autophagic pathways. Carbohyd Polym.

[CR40] Zou P, Yuan S, Yang X, Zhai X, Wang J (2018). Chitosan oligosaccharides with degree of polymerization 2–6 induces apoptosis in human colon carcinoma HCT116 cells. Chem Biol Interact.

